# The role of community pharmacists in primary and secondary prevention of skin cancer: an evaluation of a Flemish skin cancer prevention campaign

**DOI:** 10.1186/s12889-023-17429-2

**Published:** 2023-12-12

**Authors:** Kristiaan Proesmans, Frauke Van Vaerenbergh, Lies Lahousse

**Affiliations:** Faculty of Pharmaceutical Sciences, Department of Bio-analysis, Pharmaceutical Care Unit, Ghent University, Ottergemsesteenweg 460, Ghent, 9000 Belgium

**Keywords:** Skin cancer, Prevention, Community pharmacists, Sunscreen, Early detection, Counseling.

## Abstract

**Background:**

Skin cancer is a leading form of cancer in Belgium. Prevention of skin cancer by community pharmacists can play a role in increasing awareness and promoting sun protection. However, which persons could be reached by community pharmacists for skin cancer awareness in Belgium and whether this increased awareness is associated with increased sun protection and early detection remains unclear.

**Methods:**

Demographics of approached persons in Flemish community pharmacies during the months of May-June 2022 and the content of the skin cancer counseling were retrieved from the pharmacy database. Sunscreen purchases and dermatologist visits were evaluated up to 180 days after the skin cancer counseling.

**Results:**

Community pharmacists provided skin cancer counseling to a broad population of visitors (n = 822, 69% females, median age of 59 years Q1-Q3: 44–71 years). During the campaign, 822 visitors received a leaflet with skin cancer prevalence and sunscreen importance. On top of that, 335 visitors (41%) received additional counseling: skin type sensitivity was checked for 198 visitors (24%), typical characteristics of melanoma were discussed with 100 visitors (12%) and 37 visitors (5%) were referred to a physician for further information or concerns regarding a skin spot. Overall, one out of three visitors purchased sunscreen on the day of the counseling (33%, increasing up to 38% after 180 days). Among people under 20 years, this was even higher (51%). Additional counseling increased the likelihood of a dermatologist visit within 180 days (OR = 1.80; 95%CI: 1.12–2.88).

**Conclusions:**

By providing skin cancer counseling in Belgian community pharmacies, a broad range of citizens was reached and triggered to purchase sunscreen, often on the same day as the counseling. Notably, young people were likely to purchase sunscreen. Citizens receiving additional counseling were more likely to visit a dermatologist within 180 days.

## Background

Skin cancer is globally on the rise, causing a growing public health problem [1, 2]. In 2018, skin cancer (including both melanoma and non-melanoma) was the most common cancer in Belgium, with 44.000 new diagnoses [3]. Incidences are expected to further increase in the next decades [4]. Predictions say that one-fifth of the Belgian population will suffer from skin cancer before the age of 75 years [3]. Besides the public health burden, there is also a large economic burden. For the year 2014, the economic cost was estimated to be 106 million euros in Belgium [5, 6].

In contrast, skin cancer is one of the most preventable types of cancer with well-established risk factors [7–9]. A major contributor is ultraviolet (UV) irradiation. UV irradiation is linked to skin cancer, epidemiologically as well as mechanistically [2, 10–13]. Estimates showed that 86% of melanoma cases were caused by excess UV exposure [14, 15]. For non-melanoma skin cancer, the estimates also predicted a causation of over 80% [16]. Furthermore, skin cancer has a visible onset. In the early stage, the curability is over 92% and often only a simple surgical excision is required [17, 18]. Therefore prevention and early detection are considered main strategies to reduce the burden of skin cancer, improving the number of quality-adjusted life years and reducing avoidable costs of skin cancer [5, 19].

Skin cancer prevention campaigns can be divided into two main categories: primary, and secondary prevention. Primary prevention comprises education and informing on sun protection strategies to minimize harmful UV exposure. These campaigns have been shown to be able to increase sun protection behaviors [20–24]. For example, television advertising to promote the use of sunscreen and wearing hats in Australia, has enhanced protective behavior reducing sunburn by 50% [25, 26]. Also school programs have been shown to initiate small to modest behavioral changes, which potentially reduce skin cancer incidence and mortality [6, 23, 27]. Secondary prevention comprises screening and early detection of skin cancer. Currently, evidence of benefits for systematic screening for asymptomatic adults is insufficient [28, 29]. A meta-analysis of 15 studies indicates benefits of skin cancer screening programs in the adult population, but stresses the urgent need for higher-level evidence [30]. The majority of melanomas are detected through skin self-examination, showing their potential as a screening method [31]. Interventions can enhance skin self-examination activity and likely aid early detection [32].

Despite the successes of primary and secondary prevention, there are still several challenges to reach its full potential. Subpopulations show discrepancies in knowledge and risk behavior, with higher rates of skin cancer in rural and remote areas compared to the general population [33–36]. This highlights the importance of understanding the local communities, to get a broad reach [37]. Additionally, while the risks related to sun exposure are mostly known by the public, individual increased susceptible risk factors, e.g. skin type, are often unknown [33].

Among the adult population, television and print media are the main sources of information on skin health [33, 38, 39]. Furthermore, there is an increasing role of the internet, especially among the young adults [40–42]. To a lesser extent, people receive their information from healthcare professionals [38, 43]. Although it is shown that people who received information from healthcare providers have an increased knowledge of sun protection and a higher sun protective behavior, compared to people who did not receive information from a healthcare provider [33, 38, 43, 44]. Therefore, there is a raising interest in involving community pharmacists in the prevention of skin cancer [45–47]. Belgium has a dense and accessible pharmacy network [48–50]. However, it is unclear which persons could be reached by Belgian community pharmacists for skin cancer awareness. Moreover, how much this increased awareness is associated with increased sun protection and early detection remains unclear.

In 2022, the Flemish Pharmacist’s Network launched a region-wide sensitization campaign, on the prevention of skin cancer [51]. During the month of May and extended to June, Flemish pharmacists were asked to inform their visitors on how to prevent skin cancer. This comprised primary prevention by informing on sun protection strategies to minimize harmful UV exposure and secondary prevention including counseling on early signs of skin cancer.

In this study, we aim to describe the population reached and the potential impact on sun protection purchases and dermatological visits among persons who received skin cancer counseling by their community pharmacist.

## Methods

### Prevention campaign

Flemish pharmacists were invited to participate in the “Month of Prevention” campaign, dedicated to skin cancer. The prevention campaign took place in May 2022 and collection of registrations was extended to June 2022. In advance of the campaign, all the participating pharmacists were invited to a webinar for specific training. Furthermore, they were provided with posters and flyers to hand over to people visiting the community pharmacy. Pharmacists were asked to provide four different counseling actions to their visitors. First, they should inform each visitor on the main causes of skin cancer, early signs of skin cancer, and preventive actions that could be taken, together with the provision of a flyer summarizing this information. Second, the pharmacists could provide additional counseling on the prevention of skin cancer. There are three additional counseling actions they could perform. (1) The pharmacist could identify the type of skin of the visitor and advice on specific sunscreen products. (2) Specific skin spots could be assessed and additional information on the ABCDE rule, which summarize the main characteristics of melanoma, could be provided. (3) The visitor could be referred to a dermatologist, because of any concern on a suspicious-looking mole. The pharmacists were asked to register the actions that were taken, by a unique National Code Number (CNK) code.

All visitors could be approached, but special attention was asked for people who were at an elevated risk of skin cancer, e.g. light-skinned people, people who get easily sunburned, regular users of a sunbed, people with more than 50 pigment spots, people who have family members with skin cancer, people older than 50, people who had an organ transplant, people who used photosensitizing medicines, and outdoor workers.

### Data included

The data is provided by Farmaflux, a non-profit organization, collecting and processing real-time dispensing data from all community pharmacies in Belgium. Our analyses included visitors who were part of the prevention campaign. These visitors were followed up based on their purchased pharmaceutical and para-pharmaceutical products (restricted to dermatological products), registered by a unique CNK, over the period of 3 months before the visit up to 6 months after (February 1st 2022-January 1st 2023). Demographic information including age and sex of all visitors, was collected as well as purchased sunscreen products and drugs prescribed by dermatologists. In case a person received the counseling multiple times, only the first counseling was included. Because only fully de-identified anonymized aggregated claims data were used in the analyses, ethics approval was deemed unnecessary based on the national legalisation (the Belgian Personal Data Protection Act (30th July 2018–2018/40581)) and the European legalisation (GDPR (e.g. 2016/679 – art. 5 and art. 89)).

### Evaluation of the prevention campaign

The four different counseling actions are evaluated based on their potential impact on the primary and secondary prevention of skin cancer. This is done by investigating the purchase of sunscreen and the return of visitors with a dermatological prescription after 90 and 180 days of follow-up, as outcomes. Among dermatological prescriptions, the dispensing of skin cancer-related products defined as treatments for actinic keratosis or skin cancer (e.g. basal cell carcinoma) was evaluated.

### Statistical analyses

Descriptive analyses were performed to characterize people reached during the prevention campaign. Continuous variables were described by the median and the first and third quantile (Q1-Q3). Categorical variables were shown as counts (n) with percentages (%). Differences between groups were examined with a t-test for continuous variables and Chi-squared (χ²) test for categorical variables. Logistic regression was used to estimate the odds of sunscreen purchase and dermatologist visits. All analyses were performed in R software ($\normalsize {R}^{\text{\circledR }}$; version 4.2.3; Vienna, Austria) [52], using the packages dplyr and ggplot2 [53, 54], and with statistical packages of social science software (IBM SPSS $\normalsize {\text{s}\text{t}\text{a}\text{t}\text{i}\text{s}\text{t}\text{i}\text{c}\text{s}}^{\text{\circledR }}$; version 29.0.0.0; Armonk, New York, USA) [55].

## Results

### Reach of the prevention campaign

In total, 822 people received counseling regarding skin cancer prevention at the community pharmacy during the period of May-June 2022. The population approached by community pharmacists for this counseling comprised dominantly female subjects (69%). The median age was 59 years (Q1-Q3: 44–71 years). All 822 visitors received information regarding skin cancer including a flyer to increase awareness. Additional information was provided to 335 (41%) people, comprising 198 (24%) people who received specific skin information, 100 (12%) people who received information on the ABCDE rule, and 37 (5%) people who were referred to the dermatologist. The demographics are given in Table 1.

**Table 1:** Population characteristics: demographics, age, and sex, of visitors who were counseled during the prevention campaign

Characteristic	Received only preventive information	Received additional information	P-value^a^
	(n = 487)	(n = 335)	
**Age (years)**	n (%)	n (%)	0.220
< 20	32 (7)	19 (6)	
20–39	72 (15)	45 (13)	
40–64	189 (39)	144 (43)	
65–79	148 (30)	87 (26)	
≥ 80	46 (9)	40 (12)	
**Sex**	n (%)	n (%)	0.157
Female	327 (67)	237 (71)	
Male	160 (33)	98 (29)	

^a^P-value of the Chi-squared test between the distributions of the group that received preventive information and the group that received additional information

### Primary prevention: sun protection

**Fig. 1:**
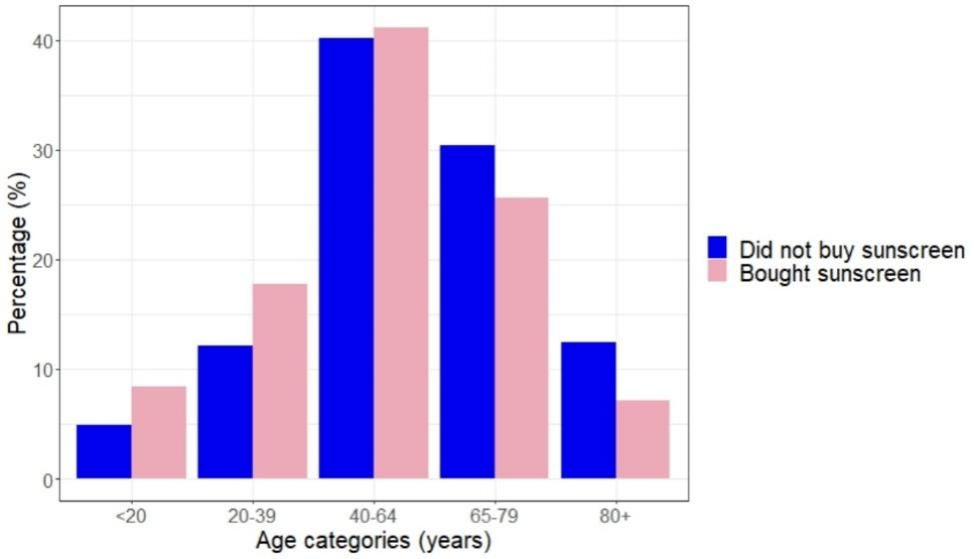
Overview of people purchasing sunscreen. The figure shows the comparison between the distribution of age groups of people who bought sunscreen and people who did not over a period of 180 days after counseling

On the counseling day, 271 (33%) visitors bought sunscreen. In the following 90 days, 33 (4%) additional people purchased sunscreen. After 180 days, 309 (38%) visitors eventually bought a sunscreen product. The age distribution among people who bought sunscreen and people who did not are shown in Fig. 1. A significant difference was observed in the distribution of age categories (χ² = 15.23, P-value = 0.004) with the youngest age group of individuals below 20 years, buying proportionally most sunscreen (51%, 95%CI: 37–65%). While the oldest age group of individuals 80 years and older, purchased the least (26%, 95%CI: 17–36%). Female visitors bought borderline significant more sunscreen than male visitors (40% vs. 33%, 95%CI: 0–14%, χ²= 3.46, P-value = 0.063).

### Secondary prevention: early skin cancer detection

In total, 92 (11%) people returned with a dermatological prescription within 180 days after the counseling (with 72 (9%) people already returning within 90 days). The chance of returning with a dermatological prescription was estimated by logistic regression modelling. Adjusted for age, sex and history of dermatological visit(s), people who received additional counseling were 2.07 (95%CI: 1.22–3.50) times more likely to return with a dermatological prescription after 90 days (Table 2). After 180 days, this was 1.80 (95%CI: 1.12–2.88). The separate counseling actions were analyzed in model 2. Skin spot assessment showed to enhance significantly the chance of returning with a dermatological prescription. This remained stable over 90 and 180 days. A referral to a dermatologist had the highest odds for a visit within 90 days, with an adjusted odds ratio of 3.47 (95%CI: 1.22–9.85). At 180 days, the effect was 2.25 (95%CI: 0.81–6.22) and was no longer statistically significant.

**Table 2:** Association between counseling and returning with a dermatological prescription

	90 days after counseling		180 days after counseling	
	*aOR*^a^ (95%CI)	P-value	*aOR*^a^ (95%CI)	P-value
**Model 1**				
Counseling	Ref.		Ref.	
Additional counseling	2.07 (1.22–3.50)	0.007	1.80 (1.12–2.88)	0.015
**Model 2**				
Counseling	Ref.		Ref.	
Skin type assessed	1.55 (0.56–2.86)	0.175	1.42 (0.81–2.51)	0.224
Skin spot assessed	2.92 (1.42–6.01)	0.004	2.53 (1.32–4.86)	0.005
Referral	3.47 (1.22–9.85)	0.020	2.25 (0.81–6.22)	0.118

^a^Covariates age (as a continuous variable), sex, and visited a dermatologist before counseling are included in the models

### Specific products to prevent and treat skin cancer

During the study period, we identified two topical products among dermatological supplies to treat or prevent further skin cancer development (e.g. fluorouracil (Efudix^\circledR^ ), and imiquimod (Aldara^\circledR^)). In total, these products were newly dispensed to 9 visitors (they did not receive these products during the 90 days before the counseling). Among these 9 people, 5 people had received additional counseling at the community pharmacy.

## Discussion

This study investigates the potential impact of community pharmacists in increasing skin cancer prevention by evaluating a sensitization campaign in Flanders. During the study period, 822 visitors were reached for skin cancer counseling at the community pharmacy. Visitors were predominantly female, which is in concordance with studies describing the visitors of community pharmacies [47, 56]. The median age was 59 (Q1-Q3: 44–71) years. The skewness towards older people could be explained by the extra attention that was asked for people older than 50 years during this campaign, because of their higher risk of skin cancer [57]. However, it remains important to address younger people as well because their awareness is lower and their risk behavior is higher [58]. Therefore, additional efforts might be necessary to reach younger ages for primary prevention. This could be done by additional prevention campaigns in for example schools or on social media [44, 59–61].

We evaluated the prevention campaign on its potential impact on short-term primary prevention and secondary preventive actions, taking into account the different additional counselling items. The first primary prevention action was estimated based on the purchase of sunscreen. Among our participants, 33% purchased a sunscreen product on the day of the counseling which only slightly increased to 38% during the 180 days follow-up period. This may indicate the importance of immediate coupling of information with action. Notably, people who bought sunscreen were generally younger. This indicates that counseling might have a bigger impact on young people and shows opportunities for the prevention of skin cancer in young people by providing accessible information. Studies show a higher general usage of sunscreen among females compared to males [62]. While in our study women were more likely to buy sunscreen, the difference was of borderline significance.

Concerning secondary prevention, 11% of the counseled people had a dermatological prescription within the first 180 days. The data suggest that people who received additional counseling on skin cancer were more likely to return with a dermatological prescription. This might indicate a higher awareness among people who received additional counseling from a healthcare professional. This is in concordance with other studies [38, 39]. Over 90 and 180 days, the effects of the additional counseling actions seemed to be stable, apart from the referral to the dermatologist, which had a slightly lower estimate at 180 days.

A separate analysis was done, to investigate the number of purchased products linked to skin cancer. From the moment of sensitization, 9 people bought a product linked to skin cancer of whom 5 people received additional information. This might show the added value of counseling in the early detection of skin cancer. However, larger studies are required.

We acknowledge several important study limitations. First, there was no control group of people who did not participate in the prevention campaign. This makes a direct comparison with people who did not receive skin cancer counseling impossible. Second, our analyses of primary prevention only include the purchase of sunscreen products at community pharmacies. We did not have information on actual use, behavior or exposure time (e.g. outdoor workers could be additionally encouraged to use sun protection when exposed to UV irradiation). Third, the analyses of the secondary prevention was limited to dermatologist visits by people returning with a dermatological prescription during the study period, while we missed all visits without (collected) prescription during that period (e.g. people who had a surgery excision in the early stage of melanoma). Finally, our analyses were limited to 180 days of follow-up. Future studies are necessary to get an overview of the broad behavioral changes and how pertinent these changes are.

Our study indicates that involving community pharmacists could play a beneficial effect both in primary as in secondary prevention of skin cancer. However, it is necessary to have broad, long term, randomized control studies to get a better view of the total impact of preventing skin cancer at community pharmacies.

## Conclusions

We evaluated the role of community pharmacists in primary and secondary prevention of skin cancer. A diverse population, primarily comprising of females and older individuals, was reached and motivated to buy sunscreen products. The purchase of sunscreen was mainly observed immediately after the consultation, indicating the benefits of giving possibilities to immediately act upon the sensitization. People who received additional counseling from pharmacists, were more likely to visit a dermatologist. This suggests that pharmacists could play an effective role in the prevention of skin cancer. However, larger randomized controlled studies need to confirm these findings.

## Data Availability

The data that support the findings of this study are available from Farmaflux but restrictions apply to the availability of these data, which were used under license for the current study, and so are not publicly available. Data are however available upon reasonable request to the corresponding author and with permission of Farmaflux.

## List of abbreviations

aOR: Adjusted odds ratio
CI: Confidence interval
Q1: First quantile
Q3: Third quantile
UV: Ultra violet

## References

[1] De Vries E, Van De Poll-Franse LV, Louwman WJ, De Gruijl FR, Coebergh JWW (2005). Predictions of Skin cancer incidence in the Netherlands up to 2015. Br J Dermatol.

[2] Leiter U, Keim U, Garbe C. Epidemiology of Skin Cancer: Update 2019. In: Reichrath J, editor. Sunlight, Vitamin D and Skin Cancer [Internet]. Cham: Springer International Publishing; 2020 [cited 2023 May 10]. p. 123–39. (Advances in Experimental Medicine and Biology). 10.1007/978-3-030-46227-7_6. 10.1007/978-3-030-46227-7_6 32918216

[3] stk_huidkanker_2021.pdf [Internet]. [cited 2023 May 10]. Available from: https://www.kanker.be/sites/default/files/stk_huidkanker_2021.pdf.

[4] Reichrath J, Sunlight. Vitamin D and Skin Cancer. Springer Nature; 2020. p. 422.

[5] Pil L, Hoorens I, Vossaert K, Kruse V, Tromme I, Speybroeck N (2016). Burden of Skin cancer in Belgium and cost-effectiveness of primary prevention by reducing ultraviolet exposure. Prev Med.

[6] Kyle JW, Hammitt JK, Lim HW, Geller AC, Hall-Jordan LH, Maibach EW (2008). Economic evaluation of the US Environmental Protection Agency’s SunWise Program: Sun Protection Education for Young Children. Pediatrics.

[7] Modifiable risk factors for cancer |. British Journal of Cancer [Internet]. [cited 2023 May 24]. Available from: https://www.nature.com/articles/6601509.

[8] Islami F, Goding Sauer A, Miller KD, Siegel RL, Fedewa SA, Jacobs EJ (2018). Proportion and number of cancer cases and deaths attributable to potentially modifiable risk factors in the United States. CA Cancer J Clin.

[9] Collatuzzo G, Boffetta P (2023). Cancers attributable to modifiable risk factors: a road map for prevention. Annu Rev Public Health.

[10] Pfeifer P, Besaratinia G (2012). UV wavelength-dependent DNA damage and human non-melanoma and Melanoma skin cancer. Photochem Photobiol Sci.

[11] Linos E, Swetter SM, Cockburn MG, Colditz GA, Clarke CA (2009). Increasing Burden of Melanoma in the United States. J Invest Dermatol.

[12] D’Orazio J, Jarrett S, Amaro-Ortiz A, Scott T (2013). UV Radiation and the skin. Int J Mol Sci.

[13] Armstrong BK, Kricker A (1993). How much Melanoma is caused by sun exposure?. Melanoma Res.

[14] Arnold M, de Vries E, Whiteman DC, Jemal A, Bray F, Parkin DM (2018). Global burden of cutaneous Melanoma attributable to ultraviolet radiation in 2012. Int J Cancer.

[15] Parkin DM, Mesher D, Sasieni P. 13. Cancers attributable to solar (ultraviolet) radiation exposure in the UK in 2010. Br J Cancer. 2011;105(2):S66–9. 10.1038/bjc.2011.486 PMC3252056 22158324

[16] O’Sullivan DE, Brenner DR, Villeneuve PJ, Walter SD, Demers PA, Friedenreich CM (2021). The current burden of non-melanoma Skin cancer attributable to ultraviolet radiation and related risk behaviours in Canada. Cancer Causes Control.

[17] Del Marmol V. Prevention and screening of Melanoma in Europe: 20 years of the Euromelanoma campaign. J Eur Acad Dermatol Venereol. 2022;36(S6):5–11. 10.1111/jdv.18195 35738812

[18] Leiter U, Buettner P, Eigentler T, Garbe C (2004). Prognostic factors of thin cutaneous Melanoma: an analysis of the Central Malignant Melanoma Registry of the German dermatological society. J Clin Oncol off J Am Soc Clin Oncol.

[19] Kornek T, Augustin M (2013). Skin cancer prevention. JDDG J Dtsch Dermatol Ges.

[20] Services UD of, H. and H. Reducing the Risk of Skin Cancer. In: The Surgeon General’s Call to Action to Prevent Skin Cancer [Internet]. Office of the Surgeon General (US); 2014 [cited 2023 May 11]. Available from: https://www.ncbi.nlm.nih.gov/books/NBK247163/.

[21] Janda M, Stoneham M, Youl P, Crane P, Sendall MC, Tenkate T (2014). What encourages sun protection among outdoor workers from four industries?. J Occup Health.

[22] Mahler HIM, Kulik JA, Gerrard M, Gibbons FX (2007). Long-term effects of appearance-based interventions on sun protection behaviors. Health Psychol.

[23] Stöver LA, Hinrichs B, Petzold U, Kuhlmei H, Baumgart J, Parpart C (2012). Getting in early: primary Skin cancer prevention at 55 German kindergartens. Br J Dermatol.

[24] A graded work. site intervention program to improve sun protection and skin cancer awareness in outdoor workers in Israel | SpringerLink [Internet]. [cited 2023 May 24]. Available from: https://link.springer.com/article/10.1023/A:1008970224998. 10.1023/a:1008970224998 10880033

[25] Saginala K, Barsouk A, Aluru JS, Rawla P, Barsouk A (2021). Epidemiol Melanoma Med Sci.

[26] Dobbinson SJ, Wakefield MA, Jamsen KM, Herd NL, Spittal MJ, Lipscomb JE (2008). Weekend Sun Protection and Sunburn in Australia: Trends (1987–2002) and Association with SunSmart Television Advertising. Am J Prev Med.

[27] Giles-Corti B, English DR, Costa C, Milne E, Cross D, Johnston R (2004). Creating SunSmart schools. Health Educ Res.

[28] Bibbins-Domingo K, Grossman DC, Curry SJ, Davidson KW, Ebell M, US Preventive Services Task Force (2016). Screening for Skin Cancer: US Preventive Services task force recommendation statement. JAMA.

[29] Johansson M, Brodersen J. Gøtzsche PC, Jørgensen KJ. Screening for reducing morbidity and mortality in malignant melanoma. Cochrane Database Syst Rev [Internet]. 2019 [cited 2023 May 25];(6). Available from: https://www.cochranelibrary.com/cdsr/doi/10.1002/14651858.CD012352.pub2/full. 10.1002/14651858.CD012352.pub2 PMC6545529 31157404

[30] Brunssen A, Waldmann A, Eisemann N, Katalinic A (2017). Impact of Skin cancer screening and secondary prevention campaigns on Skin cancer incidence and mortality: a systematic review. J Am Acad Dermatol.

[31] Hamidi R, Peng D, Cockburn M (2010). Efficacy of skin self-examination for the early detection of Melanoma. Int J Dermatol.

[32] Ersser SJ, Effah A, Dyson J, Kellar I, Thomas S, McNichol E (2019). Effectiveness of interventions to support the early detection of Skin cancer through skin self-examination: a systematic review and meta‐analysis. Br J Dermatol.

[33] Seité S, del Marmol V, Moyal D, Friedman A. j. Public primary and secondary skin cancer prevention, perceptions and knowledge: an international cross-sectional survey. J Eur Acad Dermatol Venereol. 2017;31(5):815–20. 10.1111/jdv.14104 PMC6084324 28045207

[34] Adelson P, Eckert M. Skin cancer in regional, rural and remote Australia; opportunities for service improvement through technological advances and interdisciplinary care. Aust J Adv Nurs 37(2):25–30.

[35] Glenister K, Bougoulias M, Zgibor J, Bourke L, Simmons D (2022). Self-reported skin cancer-related behaviours in rural Victoria: results from repeat cross-sectional studies in 2001–2003 and 2016–2018. Aust N Z J Public Health.

[36] Makin J, Dobbinson S, Phillips Doyle C (2009). Victorian farmers’ Skin cancer prevention knowledge and behaviours. J Occup Health Saf - Aust N Z.

[37] Montague M, Borland R, Sinclair C (2001). Slip! Slop! Slap! And SunSmart, 1980–2000: Skin Cancer Control and 20 years of Population-based campaigning. Health Educ Behav.

[38] Haluza D, Schwab M, Simic S, Cervinka R, Moshammer H (2015). Perceived relevance of educative information on public (skin) health: results of a representative, population-based telephone survey. Int J Environ Res Public Health.

[39] Haluza D, Cervinka R (2013). Perceived relevance of educative information on public (skin) health: a cross-sectional Questionnaire Survey. J Prev Med Pub Health.

[40] Krstić J, Ćorić N. Public Health Communication: Skin Cancer Prevention Implications. Manag Sustain Bus Manag Solut Emerg Econ [Internet]. 2021 Dec 11 [cited 2023 Jun 13]; Available from: http://management.fon.bg.ac.rs/index.php/mng/article/view/418.

[41] Tizek L, Schielein M, Rüth M, Szeimies R, Philipp-Dormston W, Braun S (2019). Interest in Skin Cancer in urban populations: a retrospective analysis of Google Search terms in nine large German cities. Acta Derm Venereol.

[42] Johnson KM, Jones SC, Iverson D (2009). Guidelines for the development of social marketing programmes for sun protection among adolescents and young adults. Public Health.

[43] Robinson JD, Silk KJ, Parrott RL, Steiner C, Morris SM, Honeycutt C (2004). Healthcare providers’ sun-protection promotion and at-risk clients’ skin-cancer-prevention outcomes. Prev Med.

[44] Hart KM, Demarco RF (2008). Primary prevention of skin cancer in children and adolescents: a review of the literature. J Pediatr Oncol Nurs.

[45] Pearce S, Evans A, Phelps C, Matthews M, Hughes G, Lewis I (2016). The case for targeting community pharmacy-led health improvement: findings from a skin cancer campaign in Wales. Int J Pharm Pract.

[46] Kjome RLS, Wright DJ, Bjaaen AKB, Garstad KW, Valeur M (2017). Dermatological cancer screening: evaluation of a new community pharmacy service. Res Soc Adm Pharm RSAP.

[47] Kirkdale CL, Archer Z, Thornley T, Wright D, Valeur M, Gourlay N (2020). Accessing mole-scanning through Community Pharmacy: a Pilot Service in collaboration with dermatology specialists. Pharmacy.

[48] Rondeaux S, Braeckman T, Beckwé M, Biset N, Maesschalck J, Duquet N (2022). Diabetes and cardiovascular diseases risk assessment in community pharmacies: an implementation study. Int J Environ Res Public Health.

[49] Toegankelijkheid [Internet]. [cited 2023 May 25]. Available from: https://www.apb.be/nl/corp/de-apotheker/eerstelijnszorg/Pages/toegangkelijkheid.aspx.

[50] Lijst wetten en. besluiten | FAGG [Internet]. [cited 2023 May 25]. Available from: https://www.fagg-afmps.be/nl/items-HOME/lijst_wetten_besluiten.

[51] Maand van de Preventie 2022. : Huidkanker | Vlaams Apothekers Netwerk [Internet]. [cited 2023 May 10]. Available from: https://vlaamsapothekersnetwerk.be/materialen-voor-apothekers/maand-van-de-preventie-2022-huidkanker.

[52] R: The R Project for Statistical Computing [Internet]. [cited 2023 Jun 13]. Available from: https://www.r-project.org/.

[53] A Grammar of. Data Manipulation • dplyr [Internet]. [cited 2023 Oct 11]. Available from: https://dplyr.tidyverse.org/.

[54] Villanueva RAM, Chen ZJ (2019). ggplot2: elegant graphics for data analysis. Meas Interdiscip Res Perspect.

[55] SPSS Software [Internet]. 2023 [cited 2023 Jun 13]. Available from: https://www.ibm.com/spss.

[56] Boardman H, Lewis M, Croft P, Trinder P, Rajaratnam G (2005). Use of community pharmacies: a population-based survey. J Public Health.

[57] Apalla Z, Lallas A, Sotiriou E, Lazaridou E, Ioannides D (2017). Epidemiological trends in skin cancer. Dermatol Pract Concept.

[58] 21-081142_ipsos_rapport_uv-monitor_2021_nl_v. 3.pdf [Internet]. [cited 2023 May 10]. Available from: https://www.kanker.be/sites/default/files/21-081142_ipsos_rapport_uv-monitor_2021_nl_v3.pdf.

[59] Nahar VK (2013). Skin cancer prevention among school children: a brief review. Cent Eur J Public Health.

[60] Guy GP, Holman DM, Watson M (2016). The important role of schools in the prevention of skin cancer. JAMA Dermatol.

[61] De La Garza H, Maymone MBC, Vashi NA (2021). Impact of social media on skin cancer prevention. Int J Environ Res Public Health.

[62] Falk M, Anderson CD (2013). Influence of age, gender, educational level and self-estimation of skin type on sun exposure habits and readiness to increase sun protection. Cancer Epidemiol.

